# Molecular mechanism and research progress on pharmacology of ferulic acid in liver diseases

**DOI:** 10.3389/fphar.2023.1207999

**Published:** 2023-05-31

**Authors:** Yingying Shi, Lu Shi, Qi Liu, Wenbo Wang, YongJuan Liu

**Affiliations:** ^1^ Department of Immunology, School of Medicine, Jianghan University, Wuhan, Hubei, China; ^2^ Department of Pharmacy, School of Medicine, Jianghan University, Wuhan, Hubei, China; ^3^ Department of Central Laboratory, The Affiliated Lianyungang Hospital of Xuzhou Medical University, Lianyungang, Jiangsu, China

**Keywords:** ferulic acid, liver injury, liver fibrosis, molecular mechanisms, pharmacology

## Abstract

Ferulic acid (FA) is a natural polyphenol, a derivative of cinnamic acid, widely found in Angelica, Chuanxiong and other fruits, vegetables and traditional Chinese medicine. FA contains methoxy, 4-hydroxy and carboxylic acid functional groups that bind covalently to neighbouring adjacent unsaturated Cationic C and play a key role in many diseases related to oxidative stress. Numerous studies have shown that ferulic acid protects liver cells and inhibits liver injury, liver fibrosis, hepatotoxicity and hepatocyte apoptosis caused by various factors. FA has protective effects on liver injury induced by acetaminophen, methotrexate, antituberculosis drugs, diosbulbin B and tripterygium wilfordii, mainly through the signal pathways related to TLR4/NF-κB and Keap1/Nrf2. FA also has protective effects on carbon tetrachloride, concanavalin A and septic liver injury. FA pretreatment can protect hepatocytes from radiation damage, protects the liver from damage caused by fluoride, cadmium and aflatoxin b1. At the same time, FA can inhibit liver fibrosis, inhibit liver steatosis and reduce lipid toxicity, improve insulin resistance in the liver and exert the effect of anti-liver cancer. In addition, signalling pathways such as Akt/FoxO1, AMPK, PPAR γ, Smad2/3 and Caspase-3 have been shown to be vital molecular targets for FA involvement in improving various liver diseases. Recent advances in the pharmacological effects of ferulic acid and its derivatives on liver diseases were reviewed. The results will provide guidance for the clinical application of ferulic acid and its derivatives in the treatment of liver diseases.

## 1 Introduction

**FIGURE 1:**
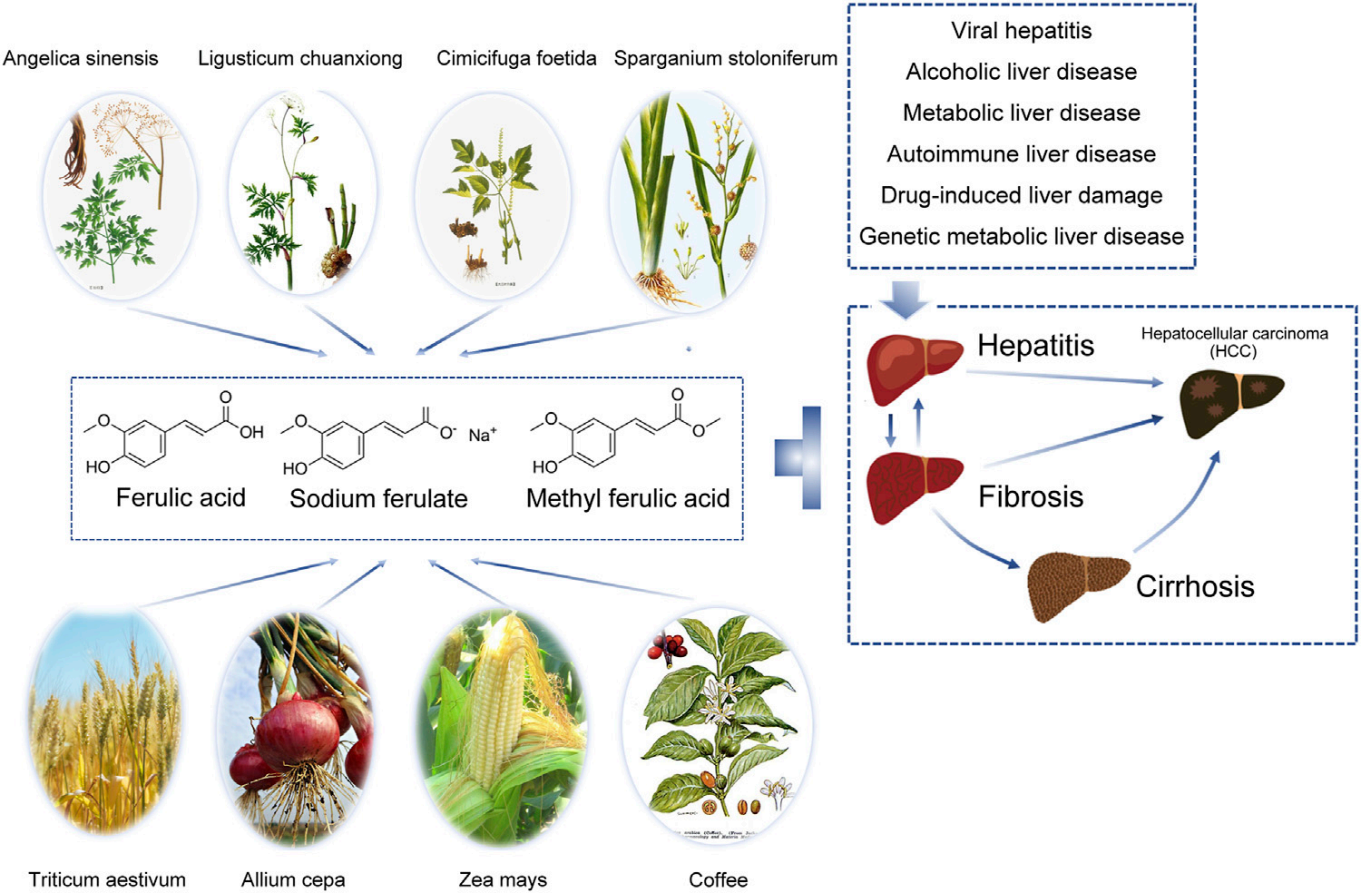
Ferulic acid and its derivatives are widely distributed and can protect the liver by inhibiting liver injury in various ways. In the hepatitis-liver fibrosis/liver cirrhosis-liver cancer trilogy, various damage factors including viral infection, toxicants and drugs, alcoholic hepatitis, non-alcoholic steatohepatitis, cholestatic cirrhosis, autoimmune liver disease, genetic metabolic diseases, *etc.*, cause hepatocyte necrosis or apoptosis, release a variety of cytokines, activate hepatic stellate cells and Kupffer cells, and lead to chronic inflammation. Liver fibrosis is the early stage of inflammatory stimulation and injury repair, which is reversible to some extent. When the pathogenic factors were removed, apoptosis occurred in activated hepatic stellate cells, collagen synthesis decreased and degradation increased, and liver structure and function recovered. If the injury and chronic inflammation persist, it can eventually develop into liver cirrhosis and liver cancer. Ferulic acid can play a protective effect on many types of liver diseases in many ways.

Ferulic acid (FA), also known as 4-hydroxy-3-methoxycinnamic acid (C_10_H_10_O_4_, MW 194.19), exists in a variety of crops and Chinese herbal medicines, and the content of FA in a crop is an important indicator of quality (Ou and Kwok, 2004). FA has multiple biological functions, such as inhibiting the release of inflammatory mediators, protecting the cardiovascular system, preventing tumours and diabetes, and alleviating neurological diseases (Li C. et al., 2021; Li D. et al., 2021; Gupta et al., 2021; Neto-Neves et al., 2021) (Figure 1).

The liver is a vital part of the digestive system and controls the functions of biochemical synthesis, metabolism, detoxification and so on. At the same time, the liver is a relatively fragile organ that is most vulnerable to biological and chemical toxins (Trefts et al., 2017). Free radicals in human body are divided into oxygen free radicals and non-oxygen free radicals (Di Meo and Venditti, 2020) (PMID: 32411336). Oxygen free radicals are dominant, accounting for about 95% of the total free radicals. Oxygen free radicals include hydrogen peroxide, hydroxyl radical, alkyl peroxy, peroxynitrite, superoxide anion O2-and so on (Di Meo and Venditti, 2020). They are collectively called reactive oxygen species (active oxygen species, ROS), which are the most important free radicals in the human body. Non-oxygen free radicals mainly include hydrogen free radicals and organic free radicals. Excessive oxygen free radicals can attack the cell membrane of liver tissue, cause lipid peroxidation and form lipid peroxides (Alkadi, 2020). Human antioxidant system is a barrier to protect the body from the damage of free radicals, delay the aging of the body and reduce the incidence of chronic diseases. The antioxidant system in the body mainly includes enzymatic reaction system and non-enzymatic reaction system (Alkadi, 2020). Enzymatic reaction system mainly refers to the antioxidant enzymes that the body is born with, such as superoxide dismutase (SOD), glutathione peroxidase (GPX), catalase (CAT) and so on. Non-enzymatic reaction system mainly refers to the intake of antioxidants from the outside through food or drugs, such as plant extracts (seabuckthorn oil, resveratrol, *etc.*), vitamins and sulfur compounds, which can control the production of free radicals in the body (Alkadi, 2020). Under normal physiological conditions, the enzymatic and nonenzymatic antioxidant systems in the body can quickly transform or eliminate free radicals, maintaining the stable state of the body. Superoxide dismutase (SOD) is a vital type of enzyme that can eliminate the excessive superoxide anion free radicals produced in the metabolic process of the body (Xu et al., 2018). Glutathione peroxidase (GSH-PX) is also a major peroxidase decomposition enzyme (Liang et al., 2011). GSH-PX activity can also reflect the degree of liver damage to some extent. Catalase (CAT), one of the key enzymes in the antioxidant defence system, can catalyse the breakdown of hydrogen peroxide and is widely present in various tissues, especially in the liver, at high concentrations (Li et al., 2018). Aspartate aminotransferase (AST) and alanine aminotransferase (ALT) are important amino transferases in the liver (Sookoian and Pirola, 2015). When liver tissue is injured, a large amount of AST and ALT are released into the blood, resulting in an increase in AST and ALT. Therefore, elevated serum AST and ALT are markers of liver injury. Malondialdehyde (MDA) is the final product of lipid peroxidation caused by free radicals (Gawel et al., 2004). The MDA content in tissue and serum can reflect the strength of lipid oxidation of the hepatocyte membrane and the degree of liver injury.

Ferulic acid, a kind of dietary flavonoid, is found in many plants such as Umbelliferae, Ranunculaceae and Gramineae, such as Angelica sinensis, Ligusticum chuanxiong, cohosh, trigonium, etc., (Babbar et al., 2021). The names, families and genera of various plants that contain FA are shown in Table 1.

**TABLE 1:** Plant source information of ferulic acid.

Plant name	Family and genus
*Angelic Sinensis* Diels	The genus of Umbelliferae
*Ligusticum chuanxiong* Hort	Umbelliferae subfamily apricinae
*Cimicifuga foetida* L	Ranunculaceae
*Sparganium stoloniferum* Buch. -Ham	Black trigonous genus of black trigonous family
*Phragmites communis* Trin	Reed of Gramineae
*Lycium chinense* Mill	Lycium barbarum L. of Solanaceae
*Notopterygium incisum* Ting ex H. T. Chang	Notopterygium of Umbelliferae
*Ferula sinkiangensis* K. M. Shen	Ferula of Umbelliferae
*Melia toosendan* Sieb. Et Zucc	Azadirachta
*Rosmarinus officinalis*	Rosemary of Labiatae
*Taraxacum mongolicum* Hand. -Mazz	Genus dandelion of Compositae
*Triticum aestivum* L	Arbor precocious grass subfamily *Triticum aestivum*
*Allium cepa*	Allium L. of Liliaceae
*Zea mays*	Zea Mays L. of the subfamily Gramineae

FA has been proven to play a major role in the treatment of various diseases (Chaudhary et al., 2019). In addition to regulating liver-related signalling pathways, FA can also play a role through its strong antioxidant and anti-inflammatory effects (Zdunska et al., 2018). FA stays in the blood longer than other dietary polyphenols and antioxidants. With the exploration and use of a new drug delivery system, the encapsulation of FA into chitosan triphosphate nanoparticles or its salt form significantly enhanced its cytocompatibility, solubility and clinical application potential (He et al., 2021; Romeo et al., 2021). Many studies have confirmed the antioxidant, anticancer, anti-inflammatory, antifibrotic, antiviral and vascular endothelial protective effects of FA. However, there is no systematic review on the relationship of FA with liver-related diseases. Therefore, we reviewed the therapeutic potential of FA in liver-related diseases and elaborated its protective mechanism in liver injury, liver fibrosis, liver cancer, hepatitis and liver lipotoxicity in detail to provide a theoretical basis for the further development and application of FA.

## 2 Protective effect on liver injury

Liver damage refers to the process of liver stress, immune response, and tissue lesions in which the liver is abnormal in liver function under the action of various pathogenic factors (physical, chemical and biological, *etc.*). Clinically, pathogen infection, alcohol abuse, drug overdose and adverse reactions, radiation damage, and industrial chemical poisons, among others, can induce pathological changes in the liver, which in turn leads to the occurrence of liver injury (Table 2).

**Table 2:** Molecular mechanisms of the pharmacological activity of ferulic acid.

Author/year	Animals or cells		Models		Dose/concentration of FA/MFA/SF		Course of treatment			Molecular mechanism	
**Protective effect on drug-induced liver injury**											
Yuan et al. (2016)	6-8 week old BALB/c mice weighting 18- 22 g		acetaminophen-induced		10, 30, or 100 mg/kg		every 8 h one time for three times within 24 h prior to APAP exposure			inhibited the expression of cytochrome P450 2E1 (CYP2E1), enhanced the activities of superoxide dismutase (SOD) and catalase (CAT) as well as the contents of glutathione (GSH);suppressed Toll-like receptor (TLR) 4 expression and dampened p38 mitogen-activated (MAPK) and nuclear factor kappa (NF-κB) activation	
			hepatotoxicity								
Wu et al. (2022)	C57BL/6J mice (22-24 g, male and female)		acetaminophen-induced acute liver injury		25, 50, 100 mg/kg		once per 12 hr for three times			associated with anti-oxidant and anti-apoptosis, regulate AMPK signal pathway and autophagy	
Roghani et al. (2020)	Male Swiss albino mice (22–25 g)		Methotrexate-Induced Hepatotoxicity		50, 100 mg/kg		once every day for one week			reduce the levels of nitric oxide (NO), malondialdehyde (MDA), interleukin-6 (IL-6), and tumor necrosis factor-α increased (TNF-α) and increase glutathione (GSH), catalase (CAT), total antioxidant capacity (TAC), superoxide dismutase (SOD), and glutathione peroxidase (GPx) content	
Mahmoud et al. (2020)	Twenty-four male Wistar rats (150–160 g) aged 7-8 weeks		a single dose of MTX at day 16		25 and 50 mg/kg		15 days			activating Nrf2/HO-1 signaling and PPARγ, and attenuating oxidative stress, inflammation, and cell death	
Yi, 2015 (in Chinese)	Male SD rats weighed 180∼220g		Liver injury model induced by antituberculous drugs in rats		30, 60, 120g/kg		8 weeks			inhibit NF-κB	
Niu et al. (2016)	Specific pathogen free male ICR mice (18-22 g body weight)		Diosbulbin B (DB) induced acute liver injury		20, 40, 80 mg/kg		once daily for 6 consecutive days			inhibiting intrahepatic inflammation and liver apoptosis	
Liu Wei, 2006 (in Chinese)	male Kunming mice, weight 18-22 g		Liver injury model of mice induced by tripterygium wilfordii glycosides		150 mg/kg		9 days			decrease ALT and AST activity	
**Protective effect on experimental liver injury**											
Cheng et al. (2018)	Sprague Dawley (SD) rats (8-10 weeks) weighing 250-300 g		Carbon tetrachloride-induced acute liver injury		25, 50 or 100 mg/kg body weight		a week			modulate the NOX4/p22^phox^/ROS-JNK/p38 MAPK signaling pathway	
Kim et al. (2011)	Male ICR mice weighing 25-30g		CCl4-induced liver injury		20, 40, and 80 mg/kg		1 h before and 2h after CCl4 injection			down-regulated the expressions of COX-2, TNF-α, TLR4, TLR2 and TLR9, and inhibited JNK, ERK and P38 signaling pathways	
Zhou Qin, 2020 (in Chinese)	Male Balb/C mice weighing 18-22g		Concanavalin A-induced immune liver injury model in mice		10, 30, 100 mg/kg		once per 8 hr for three times			inhibit the activation of CD4+T lymphocytes and the release of cytokines, reduce inflammation and apoptosis of liver tissue	
Zhong and Zhengling, 2018 (in Chinese)	BALB/C mice, male, (20 ± 2) g		D-galactosamine/lipopolysaccharide induced acute liver injury in mice		30、15、7.5 mg/kg		21 days			antioxidation	
Li Chen (2017)	adult male C57BL/6 mice, weighing 20±2 g		alcohol-induced liver oxidative injury		5, 10, 20 mg/kg		4 weeks			inhibited the expression of inflammatory factors tumour necrosis factor (TNF)- α, monocyte chemoattractant protein 1 (MCP-1), interleukin (IL)-1β and IL-6; attenuated both mRNA and protein expression of NOX4, p22phox, CYP2E1, Bax/Bcl-2; inhibited the activation of caspase 3 and 9 and downregulated the levels of p-JNK, p-p38 MAPK and p-ERK in liver.	
Cao et al. (2021a)	C57BL/6 mice (18–22 g);		cecal ligation and perforation (CLP)-induced murine ALI and lipopolysaccharide (LPS)-induced cellular ALI models		6 or 12 mg/kg;		5 days;			through the GSK-3β/NF-κB/CREB pathway	
	RAW264.7 cells				25, 50, 100 μM		24 h				
Xie et al., 2004 (in Chinese)	Male ICR mice, 16-18g		Inflammatory liver injury induced by lipopolysaccharide (LPS) combined with Bacillus Calmette-Guerin (BCG)		100 mg/kgw		10 days			inhibit the expression of adhesion molecules (ICAM-1 and e-selectin) in inflammatory tissues	
**Protective effect on liver injury induced by physical and chemical factors**											
Srinivasan et al. (2006)	primary culture of isolated rat hepatocytes		γ-irradiated and FA pretreated hepatocytes		1,5,10 ug/ml		30 min			decrease the levels of TBARS and DNA damage; increased antioxidant enzymes, GSH, Vitamins A, E and C, uric acid and ceruloplasmin levels	
Panneerselvam et al. (2013)	Male Wistar rats (130-140 g)		Fluoride-Induced Oxidative		20 mg/kg		12 weeks			reduced the degree of histological abberations and rescued lipid peroxidation	
			Hepatotoxicity								
Sanjeev et al. (2019)	male Wistar rats, weighing approximately180–190 g		cadmium-induced liver and renal oxidative damage		50 mg/kg body weight		15 and 30 days			decrease liver oxidative stress markers (MDA, LOOH, NO, TOS, PCC, OSI); inhibit inflammatory cells infiltration;reduce inflammation markers (TNF-α, COX-2, HSP-70);regulate antioxidant defense System (TTH, TSH, GSH, SOD, CAT, GPx)	
Wang Z et al. (2021)	Six-week-old male Wistar rats		aflatoxin B1-induced liver injury		120 mg/kg		30 days			Inhibiting cytochrome P450 enzyme, activating Nrf2/GST pathway and regulating mitochondrial pathway	
**Inhibition of liver fibrosis**											
Xu et al. (2015)	HSC-T6 cells		Hepatic stellate cells (HSCs)		3–30 uM		0-72h			Blocking transforming growth factor-b by a neutralizing antibody caused a marked reduction in both ERK1/2 and Smad signaling	
Wang Z et al., 2021 (in Chinese)	Male SD rats (6 weeks, 180-220 g)		Carbon tetrachloride (CCl4) -induced liver fibrosis model		15, 30mg/kg		6 weeks			inhibited mitogen-activated protein kinase (MAPK) signaling pathway and NF-κB/IκBα pathway	
Wu et al. (2021)	C57BL/6J mice (22–24 g, male and female, SPF grade); RAW 264.7 cells and LX-2 cells, Mouse primary hepatocytes (MPHs)		carbon tetrachloride (CCl4)-induced chronic inflammation and liver fibrosis		FA (25, 50 and 100 mg/kg); FA or MF (both 50, 100, and 200 μM), FA or MF (both 12.5, 25 and 50 μM)		4 weeks;			through PTP1B-AMPK signaling pathways	
							24 h				
Li Chen, 2017 (in Chinese)	Male SD rats, body weight 180-220 g		Carbon tetrachloride (CCl4) -induced liver fibrosis rat model		20、10、5 mg/kg		2 w			improve the oxidative stress injury of liver and reduce the accumulation of extracellular matrix	
Guo Ling, 2019 (in Chinese)	Rat hepatocytes BRL		Transforming growth factor-β (TGF-β) -induced hepatocyte injury		100, 200, 400 μmol		12 h			inhibit the expression of Smad3 induced by TGF-β1, promote the expression of MMP-2 and MMP-9, and reduce the apoptosis of hepatocytes	
Mu et al. (2018)	Human hepatic stellate cell line (HSC) LX-2;Male Wistar rats (180±20g)		CCl4-induced rat liver fibrosis		30 µM		8 weeks			inhibit the TGF-β1/Smad pathway in vitro and in vivo	
					10 mg/kg						
Cheng et al. (2019b)	Male Sprague-Dawley (SD) rats (180 ± 20 g); Human hepatic stellate cells (LX-2)		CCl4 induced liver fibrosis		MFA (20 and 5 mg/kg); 5, 10, 20 μM		5 weeks;			inhibiting the TGF-β1/Smad and NOX4/ROS signalling pathways	
							48 h				
Xiong Meili, 2016 (in Chinese)	Human hepatic stellate cell line HSC-LX-2		Transforming growth factor (TGF) -β1 stimulates human hepatic stellate cell (HSCS) -LX-2		1.25、2.5、5 mg/L		72h			decreased the expression of α -SMA and PC I, and inhibited the synthesis of HSC-LX-2 extracellular matrix and phenotype transformation of HSC-LX-2	
Zhao H et al. (2020)	Male Wistar rats (200-230 g)		Bile Duct-Ligated Cirrhotic Rats		FA (4.8, 10.8 mg/kg)		28 days			inhibition of the TGF-β pathway and activation of the Nrf2 pathway	
Juan Yang, 2014 (in Chinese)	Wistar rats, 180-200 g		Experimental hepatic fibrosis model induced by bile duct ligation in rats		50 mg/kg		1 week			decrease the expression of α-SMA in liver	
Liu et al. (2015)	Male Wistar rats with approximate body weight of 180–200 g		Bile duct ligation method was used to induce cirrhosis		50 mg/kg/day		1 week			inhibits hepatic RhoA/Rho-kinase signaling and activates the NO/PKG pathway	
Wei et al. (2014)	Male Wistar rats (180–200 g; n=73)		bile duct ligation (BDL) model of portal hypertension		50 mg/kg/day		1 week			inhibits the activation of the RhoA/Rho-kinase signaling pathway	
Hussein et al. (2020)	male albino Wistar rats weighing from 180 to 220 g		thioacetamide-induced fibrosis		20 mg/kg		6 weeks			inhibit TGF-β1/Smad3 signaling and differentially regulate the hepatic expression level of miR-21, miR-30 and miR-200	
**Inhibiting liver steatosis and reducing lipid toxicity**											
Xu et al. (2021)	AML-12, a nontransformed mouse hepatocyte cell line		AML-12 mouse hepatocytes were exposed to palmitate to mimic lipotoxicity		25, 50, and 100 μM		2h			regulate the SIRT1/autophagy pathway	
Cheng et al. (2018)	Human hepatocyte L-02 cells (human normal liver cells; no		ethanol-induced hepatic steatosis		12.5, 25, 50, 100, 200, 400 μM		24 h			activate AMPK-ACC/MAPK-FoxO1 pathway and up-regulating the expression levels of SIRT1, PPAR-α, and CPT-1α	
	GDC079); Sprague-Dawley (SD) rats weighing 180–220 g				5, 10, 20mg/(kg day)		16 weeks				
Wang X et al. (2021)	C57BL/6 mice (male, 4-week-old); HepG2 cells		oleic acid (OA)-treated HepG2 cells and C57BL/6 mice fed a high fat diet (HFD)		FA (20 mg/kg bw⋅day); FA (25 and 50 μg/mL)		17 weeks; 24 h			suppress of ERK1/2, JNK1/2/3, and HGMB1 expression	
**Improving insulin resistance in the liver and anti-liver cancer**											
Cheng et al. (2019b)	Male Sprague-Dawley (SD) rats (180–220 g); Human hepatocyte L-02 cells		Ethanol-Induced Hepatic Insulin		MFA (5, 10, 20 mg/kg/day); MFA (20 mg/kg/day)		4 weeks;			activated the hepatic phosphatidylinositol 3-kinase (PI3K)/AKT pathway	
			Resistance				24h				
Zhang et al. (2022)	Four-week-old male C57BL/6J mice		alcohol-induced hepatic insulin resistance		5, 10, 20 mg/kg		2 weeks			attenuate the inhibition of miR-378b on IR/p110α and activate the insulin signaling	
Ezhuthupurakkal et al. (2018)	Huh-7 and HepG2 cells;		hepatocellular cancer		0.05, 0.1, 1, 5, 10 and 20 μg/ml;		24 h			promote intracellular ROS generation, induce DNA damage, promote cell apoptosis	
	Wistar rats of 4-5 weeks old and weighing 80-120 g				5mg/kg/b.wt		4 weeks				
Das et al. (2019)	The HepG2, A549, CT26 and WI-38 cells		lung (A549) and liver (HepG2) carcinoma cells by treatment with ferulic acid (FA) prior to irradiation		10-400 mM		6 h, 72 h			collapse redox homeostasis and regulate Akt/p38 MAPK signaling pathway	
**Other mechanisms**											
Kim and Lee (2012)	Male ICR mice (24–26 g)		ischemia/reperfusion-induced hepatocyte apoptosis		50, 100, 200mg/kg		30 min			inhibit JNK activation and apoptosis	
RUKKUMANI et al. (2012)	Male Wistar albino rats strain of body weight ranging 140-160g		alcohol and PUFA induced toxicity		20 mg/kg body weight		45 days			decreased the levels of collagen, tissue inhibitors of metalloproteinases (TIMPs) and promote the expression of matrix metalloproteinases (MMPs).	
WANG et al. (2014)	ICR male mice (18–22 g)		diosbulbin B-induced		8 mg/kg		12 d			increase the enzymatic activities of CuZn-SOD and CAT	

### 2.1 Protective effect on drug-induced liver injury

Drug-induced liver injury (DILI) refers to liver injury caused by toxic side effects or allergic reactions caused by drugs and their metabolites (Andrade et al., 2019). In recent years, the incidence of DILI has been increasing and has attracted increasing attention. Studies have found that antibiotics, anti-inflammatory drugs, antidepressants and many other common types of drugs can cause DILI.

#### 2.1.1 Liver injury induced by paracetamol (acetaminophen)

Acetaminophen (APAP), also known as paracetamol, is a widely used antipyretic analgesic and anti-inflammatory drug (Ramachandran and Jaeschke, 2019). APAP is safe and effective at therapeutic doses, but overuse or long-term use can cause liver injury (Lee, 2017).

**FIGURE 2:**
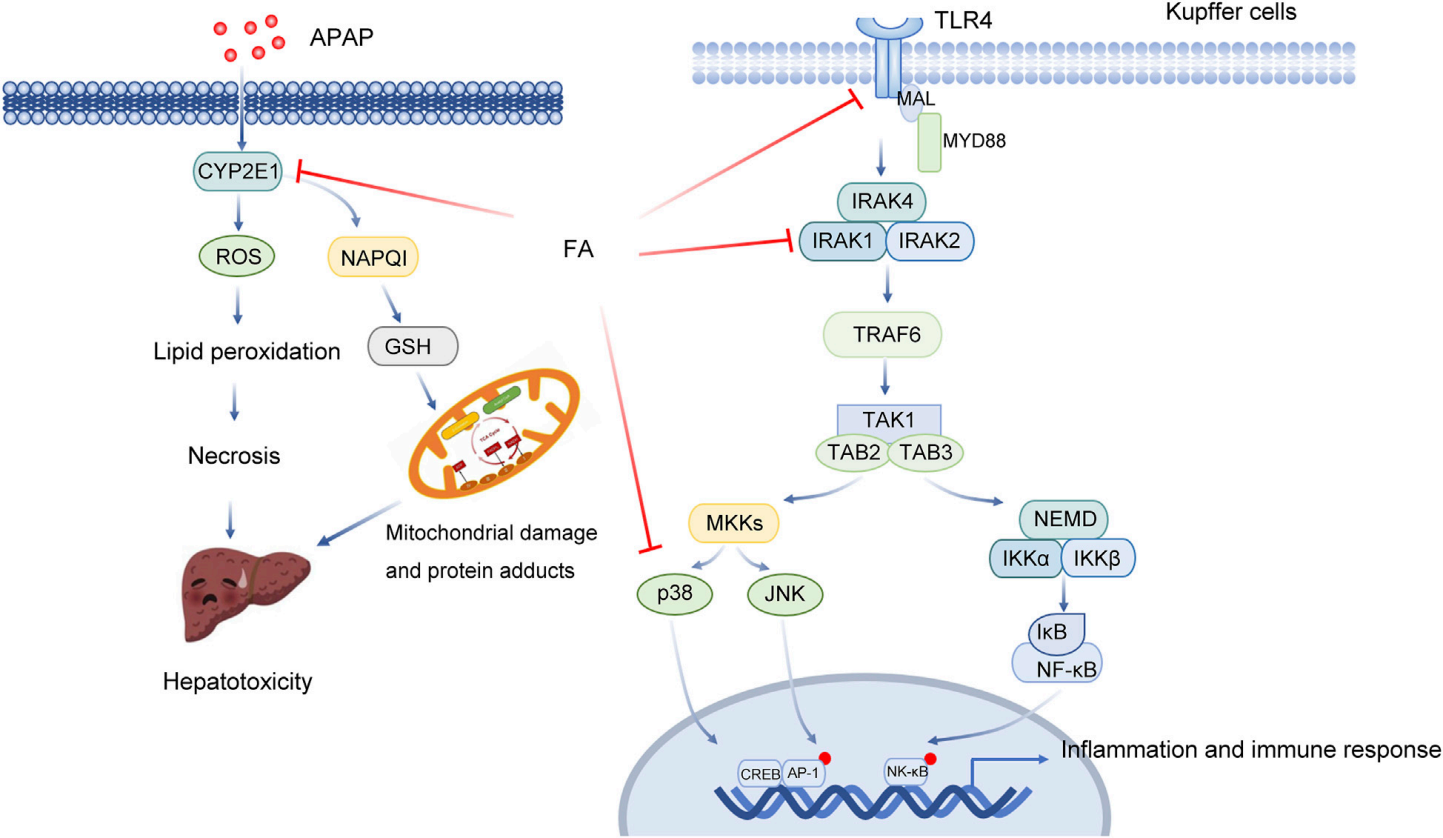
Multitarget role of ferulic acid in Apap-mediated hepatotoxicity. Abbreviations: APAP, acetaminophen; CYP2E1, Cytochrome P450 Family 2 Subfamily E Member 1; ROS, Reactive Oxygen Species; NAPQ1, NAD(P)H Quinone Dehydrogenase 1; GSH, glutathione, r-glutamyl cysteingl + glycine; TLR4, Toll Like Receptor 4; MAL, Mal, T Cell Differentiation Protein; MYD88, MYD88 Innate Immune Signal Transduction Adaptor; IRAK4, Interleukin 1 Receptor Associated Kinase 4; IRAK1, Interleukin 1 Receptor Associated Kinase 1; IRAK2, Interleukin 1 Receptor Associated Kinase 2; TRAF6, TNF Receptor Associated Factor 6; TAK1, Mitogen-Activated Protein Kinase Kinase 7; TAB2, TGF-Beta Activated Kinase 1 (MAP3K7) Binding Protein 2; TAB3, TGF-Beta Activated Kinase 1 (MAP3K7) Binding Protein 3; MKKs, Mitogen-Activated Protein Kinase; JNK, Mitogen-Activated Protein Kinase 8; NEMO, Inhibitor Of Nuclear Factor Kappa B Kinase Regulatory; IKK, Inhibitor Of Nuclear Factor Kappa B Kinase; IκB, Inhibitor Of Nuclear Factor Kappa B Kinase; NF-κB, Nuclear Factor Kappa B; CREB, CAMP Responsive Element Binding Protein; AP-1, Jun Proto-Oncogene, AP-1 Transcription Factor Subunit.

At the therapeutic dose, 90% of APAP is metabolized through the liver and binds to sulfuric acid or glucuronic acid to be excreted (Ramachandran and Jaeschke, 2019). Approximately 5%–9% of them are metabolized by cytochrome P450 oxidase (CYP450, including CYP2E1, CYP1A2, CYP2A6, CYP2D6 and CYP3A4) to produce highly active N-acetyl-p-benzoquinone imine (NAPQI), which covalently binds to GSH and is inactivated and excreted through the kidney (Ramachandran and Jaeschke, 2019). After taking too much APAP, the metabolic pathway of glucuronic acid or sulfuric acid is saturated, and a large amount of APAP is oxidized by CYP450, resulting in rapid depletion of GSH. At this time, the remaining NAPQI covalently binds to hepatocyte proteins (especially mitochondrial proteins), resulting in oxidative stress, mitochondrial dysfunction, DNA damage and other toxic reactions and finally leading to hepatocyte apoptosis and necrosis, which is the initial stage of liver injury. It is generally believed that mitochondrial oxidative stress is the main event of APAP-induced acute liver injury. Subsequently, dead hepatocytes release a large number of damage-related molecular patterns (DAMPs) (Minsart et al., 2020). Studies have shown that high mobility group protein B1 (HMGB1) and mitochondrial DNA (mtDNA) are the main DAMPs released from the damaged liver induced by APAP (Minsart et al., 2020). HMGB1 released by damaged liver cells can be recognized by TLR4 on the surface of macrophages and activates downstream signalling pathways. Activated immune cells then release inflammatory cytokines and chemokines and recruit peripheral immune cells into the liver, thus initiating the inflammatory cascade reaction. Cascade-activated immune responses cause secondary damage to hepatocytes and result in a large amount of hepatocyte death. Knockout of TLR4 or the use of TLR4 antagonists reduced APAP-induced hepatotoxicity. In addition, inflammation, apoptosis and autophagy may also play an important role (Table 2; Figure 2) (Chao et al., 2018).

FA can reduce APAP-induced hepatotoxicity by downregulating both CYP2E1 expression and TLR-4 signal pathway activation. Junhui Yuan et al. found that FA decreased the levels of ALT and AST and reduced liver apoptosis (Yuan et al., 2016). Haematoxylin-eosin (HE) staining results showed that FA reduced the degree of necrosis and bleeding, alleviating the pathological changes caused by APAP. The results of TUNEL staining and Caspase-3 activity detection showed that the degree of apoptosis decreased significantly in the FA pretreatment group. FA significantly inhibited the mRNA and protein expression of CYP2E1 and suppressed oxidative stress. After the intervention of FA, both the content of GSH in liver tissue and the activity of SOD and CAT increased significantly. FA significantly decreased the expression of TNF-α and IL-1βand downregulated the protein expression of TLR-4, p-IRAK1 and p-p38 induced by APAP (Yuan et al., 2016). Meanwhile, FA can inhibit the secretion of inflammatory factors and mediate the anti-inflammatory effect.

FA could alleviate the damage caused by APAP by activating AMPK and inducing autophagy. In another study, an APAP-induced acute liver injury (ALI) mouse model was established. It was found that APAP treatment could lead to liver necrosis (Wu et al., 2022). APAP can cause a significant increase in serum ALT, AST and oxidative stress. However, the liver injury of mice in the FA treatment group was significantly relieved. FA substantially reduced the activities of ALT, AST and MDA and increased the level of SOD. FA also suppressed liver cell necrosis and inflammatory cell infiltration. FA increased the expression of hepatocyte nuclear factor 4 alpha (HNF4a), forkhead box A2tbox2 (Foxa2) and albumin, which are important molecules in liver cells. FA further inhibited APAP-induced hepatocyte apoptosis by increasing Bcl-2 and decreasing caspase-3 and Bax protein levels. The results of functional enrichment suggest that FA is involved in the regulation of the mitogen-activated protein kinase (MAPK) signalling pathway and other metabolic processes (Wu et al., 2022).

#### 2.1.2 Liver injury induced by methotrexate (MTX)

**FIGURE 3:**
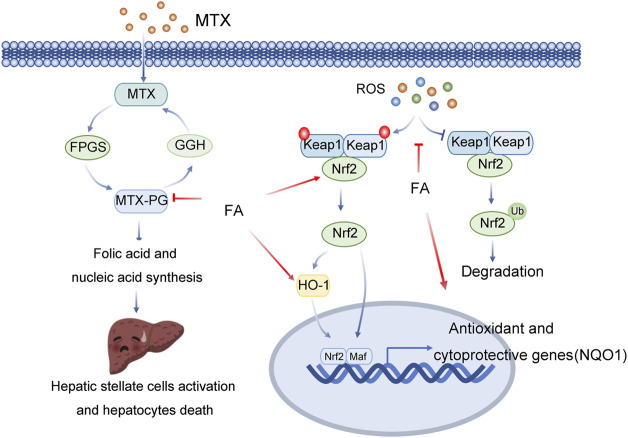
Multitarget role of ferulic acid in MTX-mediated hepatotoxicity. Abbreviations: MTX, Methotrexate; GGH, Gamma-Glutamyl Hydrolase; FPGS, Folylpolyglutamate Synthase; MTX-PG, Mtx-polyglutamate; ROS, Reactive Oxygen Species; Keap1, Kelch Like ECH Associated Protein 1; Nrf2, NFE2 Like BZIP Transcription Factor 2; HO-1, Heme Oxygenase 1; Maf, MAF BZIP Transcription Factor; NQO1, NAD(P)H Quinone Dehydrogenase 1.

Methotrexate (MTX) is a commonly used drug in the clinical treatment of skin diseases and autoimmune diseases (Rihacek et al., 2015). MTX is also a folic acid antagonist and can be used as an anticancer drug. However, long-term use of MTX is likely to cause the accumulation of metabolites in the liver, leading to liver damage (Rihacek et al., 2015). Mtx-polyglutamate (MTX-PG) is one of the most important metabolites that causes liver injury (Ezhilarasan, 2021). MTX-PG can induce oxidative stress in the liver by inducing lipid peroxidation and promote the release of inflammatory factors (Ezhilarasan, 2021). At the same time, MTX-PG inhibits the synthesis of 5-imidazole-4-carboxamide ribonucleotide converting enzyme and folic acid and reduces the synthesis of intracellular nucleic acids, resulting in the activation of hepatic stellate cells (HSCs) and liver cell death (Table 2; Figure 3). Mozhdeh Roghani et al. established a drug injury model by intraperitoneal administration of methotrexate (20 mg/kg) in mice (Roghani et al., 2020). The expression levels of liver biochemical markers, oxidative stress markers and proinflammatory cytokines were further measured. The results showed that ALT, AST, ALP, MDA, nitric oxide (NO), TNF-α and IL-6 were significantly increased in the model group, while the expression levels of GPx, SOD, CAT, GSH and TAC were significantly decreased. All of the above indicators were reversed in the FA treatment group, with reduced activation and damage to inflammatory cells and liver cells in mouse tissues compared to the model group (Roghani et al., 2020).

FA can reduce oxidative stress, inflammation and cell death by activating Nrf2/HO1 signalling and PPAR-γ, preventing methotrexate-induced hepatotoxicity. The nuclear factor E2-related factor 2 (Nrf2) signalling pathway is the main antioxidant mechanism in eukaryotic cells (Bellezza et al., 2018). Nrf2 is commonly present in the cytoplasm and interacts with Kelch-like Ech-associated protein 1 (Keap1) (Bellezza et al., 2018). Keap1 is the adaptor of the cullin-3 (Cul3) ubiquitin ligase. In the resting state, Keap1 binds Nrf2 and promotes Nrf2 ubiquitination and proteasome degradation through Cul3 ubiquitin ligase complexes. After activation, Nrf2 is translocated into the nucleus and binds with antioxidant response elements (AREs) to promote the transcription of various antioxidant and detoxification enzyme genes, including haem oxygenase-1 (HO1, also known as HMOX1), NAD(P) H-quinone oxidoreductase 1 (NQO1), Y-glutamyl cysteine ligase catalytic subunit (GCLC) and Y-glutamyl cysteine ligase modified subunit (GCLM), to combat oxidative damage (Bellezza et al., 2018). Ayman M. Mahmoud et al. found that FA prevents a variety of histological changes caused by methotrexate (MTX), including steatosis, inflammatory cell infiltration and bleeding (Mahmoud et al., 2020). FA also induces changes in serum transaminase, bilirubin and albumin. FA improves liver function, inhibit oxidative stress and enhances the expression level of antioxidants. FA decreased serum TNF-α and IL-1β, decreased NF-κBp65, Bax and caspase-3 in liver tissue, and increased Bcl-2, Nrf-2, NQO1, HO-1 and PPAR-γ. Therefore, based on the above studies, (Mahmoud et al., 2020).

#### 2.1.3 Liver injury induced by antituberculosis drugs

Tuberculosis is an ancient and well-known disease that still threatens human health (Zhao X M et al., 2020). Tuberculosis treatment requires a combination of drugsand long-term continuous treatment which is prone to cause liver damage. Isoniazid is the main tuberculosis drug causing liver toxicity, and most other reported drug-induced liver injuries are associated with isoniazid (Zhao H et al., 2020). In one study, SD male rats were treated with an intraperitoneal injection of isoniazid 70 mg/kg and intragastric administration of rifampicin to establish an animal model of liver injury induced by antituberculosis drugs (Yi, 2015). Serum levels of ALT, AST, IL-1β, IL-6 and TNF-α were significantly lower in the medium- and high-dose sodium ferulate intervention groups than in the model group. The protein expression of TNF-α and NF-κB was also decreased in the liver. It is suggested that sodium ferulate has a protective effect on liver injury induced by anti-tuberculosis drugs by inhibiting the expression of NF-κB (Yi, 2015).

#### 2.1.4 Liver injury induced by diosbulbin B

Diosbulbin B (DB) is a diterpene lactone extracted from Dioscorea bulbifera L (DBL). That has antitumour activity (Li C. et al., 2021; Li D. et al., 2021). The liver is the main toxic target organ of diosbulbin B (Ma et al., 2014). It has been reported that FA can prevent acute liver injury caused by diosbulbin B by inhibiting intrahepatic inflammation and hepatocyte apoptosis (Niu et al., 2016). Histological evaluation showed that FA (80 mg/kg) reduces hepatocyte degeneration and lymphocyte infiltration induced by diosbulbin B (Niu et al., 2016). TUNEL staining showed that FA (80 mg/kg) reduced hepatocyte apoptosis (Niu et al., 2016). FA (40, 80 mg/kg) reduced the content of MDA in the liver induced by diosbulbin B, the levels of TNF-α and IFN-γ in serum and the activity of myeloperoxidase in liver tissue induced by diosbulbin B (Niu et al., 2016). FA (80 mg/kg) reversed the downregulated expression of kappa B inhibitor (IκB) and promoted nuclear translocation of NF-κBp65 induced by diosbulbin B (Niu et al., 2016).

#### 2.1.5 Liver injury induced by tripterygium wilfordii

Polyglycosides of Tripterygium wilfordii (Tripterygium Glycosides) are extracted from Tripterygium wilfordii and are often used to treat rheumatoid arthritis, chronic glomerulonephritis, systemic lupus erythematosus and other diseases (Zhang et al., 2021). Clinical studies have shown that Tripterygium wilfordii polyglycosides can cause liver injury. In one study, the liver injury model of mice induced by polyglycosides of Tripterygium wilfordii was established by continuous treatment with Tripterygium wilfordii polyglycosides 1.8 g/(kg·d) for 6 days, and the effect of sodium ferulate on the injury was explored (Liu Wei, 2006). Experimental results indicated that the liver index and serum ALT increased significantly in the model group, while the serum ALT and AST activities decreased significantly in the sodium ferulate group, suggesting that sodium ferulate exerted a protective effect (Liu Wei, 2006).

### 2.2 Protective effect on experimental liver injury

#### 2.2.1 Liver injury induced by CCL4

Carbon tetrachloride (CCl4) is widely used to induce liver injury in animal models (Unsal et al., 2021). CCl4 produces highly active free radicals mediated by cytochrome P450. CCl4-induced liver injury is characterized by an inflammatory reaction, the formation of trichloromethyl radicals and excessive production of ROS(Unsal et al., 2021). These free radicals eventually lead to hepatotoxicity. Anti-apoptosis, antioxidation and anti-inflammation may play vital roles in protecting the body from CCl4-induced liver injury.

MFA alleviates CCl4-induced apoptosis of liver cells through the JNK and P38 MAPK signalling pathways, reducing necrosis of hepatocytes and infiltration of inflammatory cells. Chengfang Yang et al. constructed a liver injury model using CCl4(Yang et al., 2018). It was found that serum ALT, AST and liver tissue MDA decreased significantly, while liver tissue SOD and GSH-Px increased significantly in the methyl ferulic acid (MFA) group (50 mg/kg, 100 mg/kg, 200 mg/kg). The results of liver pathological sections showed that MFA improved the pathological injury of the liver. Western blotting and RT-PCR indicated that MFA effectively inhibited the expression of NOX4 and Caspase-3 in the liver. MFA has a strong quenching effect on ROS and can reduce the peroxidation damage of hepatocytes. MFA alleviated liver injury by downregulating the NOX4/ROS-p38 MAPK signalling axis and alleviating the damage caused by oxidative stress. Meanwhile, MFA exerts a protective effect by reducing the levels of aspartate-specific cysteine protease-3 in hepatocytes and inhibiting hepatocyte apoptosis. MFA suppresses the expression of the inflammatory factors TNF-α and IL-1β(Yang et al., 2018). In another study, it was reported that FA could significantly attenuate the increase in serum transaminase activity and liver MDA levels after CCl4 treatment and reduce cyclooxygenase-2 and nitric oxide synthase levels (Kim et al., 2011). In addition, FA markedly suppressed the levels of p-JNK, p-p38 and TLR4 and exerted a protective effect by inhibiting inflammation and oxidative damage (Kim et al., 2011).

#### 2.2.2 Immune liver injury induced by concanavalin A

Concanavalin A-induced liver injury is an immune liver injury caused by T-cell-mediated cytokine imbalance and can mimic the pathologic characteristics of multiple viral and autoimmune hepatitis (Ibrahim et al., 2021). Concanavalin A-induced liver injury model is a widely used model of immune liver injury. Zhou Qin et al. established a model of immune liver injury by using concanavalin A and intervened with FA (Zhou Qin, 2020). The results showed that FA inhibited the activation of CD4^+^ T lymphocytes and reduced the release of inflammatory cytokines (TNF-α and IFN-γ). FA inhibits the activation of caspase-3, which is a key molecule in apoptosis, and reduces liver tissue necrosis. FA not only inhibited local inflammation in tissues but also reduced apoptosis injury in liver cells, restored normal levels of ALT and AST in serum, and alleviated immune liver injury (Zhou Qin, 2020).

#### 2.2.3 D-galactosamine/lipopolysaccharide-induced acute liver injury

Lipopolysaccharide is the most important pathogenic agent of G-bacteria, causing the release of inflammatory factors and leading to an overactive systemic inflammatory response and tissue damage (Park and Lee, 2013; Zhou Qin, 2020). D-galactosamine is an interfering agent of uracil phosphate in hepatocytes, which can cause necrosis and inflammation of hepatocytes (Yang et al., 2022). D-galactosamine was found to increase the inflammatory effect of lipopolysaccharide. Therefore, D-galactosamine/lipopolysaccharide can be used to establish a model of acute liver injury (Yang et al., 2022). Zhengling Zhong et al. found that compared with the D-galactosamine/lipopolysaccharide model group, the liver index and serum ALT and AST levels of mice in the high-, medium- and low-dose FA groups decreased (Zhong Zhengling, 2018). The activity of SOD and GSH-Px in liver tissue increased, while the level of MDA decreased to different degrees. The results of liver pathological examination showed that the high-, medium- and low-dose groups of FA could reduce the degeneration and necrosis of hepatocytes to varying degrees (Zhong Zhengling, 2018).

It is generally believed that tumour necrosis factor α (TNF-α) is not only an important immune response molecule but also an important inflammatory mediator with a wide range of biological effects (Zelova and Hosek, 2013). Adhesion molecules are important membrane proteins that mediate the contact and binding between cells and the extracellular matrix. These molecules play a major role in the maintenance of normal tissue structure, wound healing and other pathophysiological processes. Among them, ICAM-1 and E-selectin are particularly important. In one study, a mouse model of acute inflammatory liver injury was established by lipopolysaccharide combined with *Bacillus* Calmette Guerin (BCG) (Xie et al., 2004). Experimental results showed that sodium ferulate significantly inhibited lipopolysaccharide-induced liver inflammation by regulating the expression of E-selectin and ICAM-1 in inflammatory tissue (Xie et al., 2004).

#### 2.2.4 Liver injury caused by alcohol

The liver is the dominant target organ of ethanol toxicity (Louvet and Mathurin, 2015). Increased alcohol intake will increase its metabolism through the nicotinamide adenine phosphate dinucleotide-dependent CYP2E1 oxidation pathway, leading to excessive production of ROS(Louvet and Mathurin, 2015). Excessive alcohol intake can trigger a variety of liver injury events, including cell membrane phospholipid peroxidation. It has been reported that FA has a protective effect on ethanol- and polyunsaturated fatty acid-induced hepatotoxicity by regulating the activities of liver iconic enzymes, such as alkaline phosphatase, glutamyl transaminase, glutamic pyruvic transaminase and AST (Li et al., 2017). Chen Li et al. established a liver injury model by intragastric administration of alcohol into C57BL/6 mice (Li et al., 2017). Further studies on serum transaminase activity, ADH and ALDH levels in the supernatant of liver tissue homogenate, lipid peroxidation levels of liver tissue samples, liver lipid metabolism levels (TG, TC, LDL and HDL) and liver inflammatory cytokine levels (TNF-α, McP-1, IL-1β and IL-6) were conducted. The results showed that MFA reduced alcohol-induced oxidative stress by downregulating NOX4, P22Phox, MDA, ROS, ALT and AST levels. MFA alleviates oxidative stress metabolic disorder induced by alcohol by reducing CYP2E1 levels and increasing ADH and ALDH levels. In addition, MFA inhibits the expression and activation of apoptosis-related proteins (Bax/Bcl-2, caspase-3/9) and inhibits the expression of inflammatory factors. NOX4/ROS and MAPK signalling pathways were found to be involved in the MFA-mediated liver protective effect (Li et al., 2017).

#### 2.2.5 Septic liver injury

**FIGURE 4:**
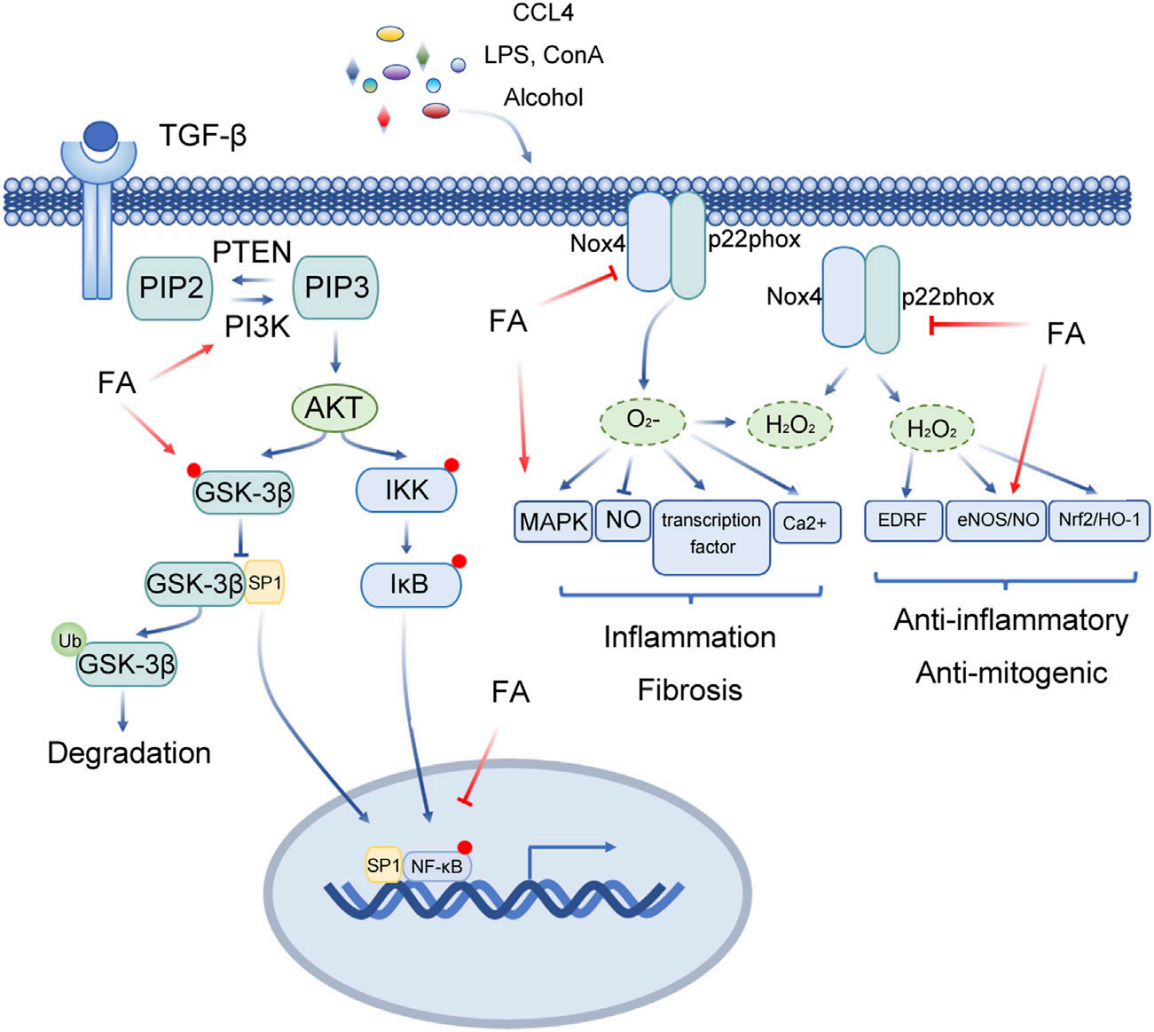
Multitarget role of ferulic acid in Liver injury mediated by other factors. Abbreviations: FA, Ferulic acid; CCL4, carbon tetrachloride; LPS, lipopolysaccharide; Con A, concanavalin A; TGF-β, Transforming Growth Factor Beta 1; PIP2, phosphati-dylinositol-4,5-bisphosphate; PTEN, Phosphatase And Tensin Homolog; PIP3, Phosphatidylinositol 3,4,5-trisphosphate; PI3K, Phosphatidylinositol-4,5-Bisphosphate 3-Kinase; AKT, Protein Kinase B; IKK, Inhibitor Of Nuclear Factor Kappa B Kinase; IκB, Inhibitor Of Nuclear Factor Kappa B Kinase; GSK-3β, Glycogen Synthase Kinase 3 Beta; SP1, Sp1 Transcription Factor; Nox4, NADPH Oxidase 4; P22phox, Cytochrome B-245 Alpha Chain; MAPK, Mitogen-Activated Protein Kinase; NO, Nitric Oxide; EDRF, Alpha Hemoglobin Stabilizing Protein; eNOS, Nitric Oxide Synthase 3; Nrf2, NFE2 Like BZIP Transcription Factor 2; HO-1, Heme Oxygenase 1.

The mortality rates of sepsis and septic shock are high, and the main cause of death is secondary multiple organ dysfunction. Glycogen synthase kinase 3 (GSK3) regulates inflammation and cytokine expression by regulating the activity of a variety of transcription factors(Cao et al., 2021a; Cao et al., 2021b). In general, the function of GSK3 depends on the phosphorylation form of tyrosine residues (GSK3α is Tyr279, GSK3β is Tyr216). When serine residues are phosphorylated (GSK3α is Ser21, GSK3 β is Ser9), the activity of GSK3 is inhibited. Liping Cao et al. confirmed that FA pretreatment markedly reduces the ratio of liver to body weight, decreases the activities of MPO, AST and ALT, reduces inflammatory reactions, and improves the histopathological changes in the liver induced by caecal ligation and perforation (CLP) (Cao et al., 2021a; Cao et al., 2021b). The results showed that FA increases the viability of RAW264.7 cells and reduce proinflammatory cytokines. FA promotes the expression of p-GSK-3β and CREB protein, decreases the level of p-NF-κB, improves the inflammatory response induced by acute liver injury (ALI) through the GSK-3β/NF-κB/CREB pathway, and protects the liver from acute liver injury (ALI) (Cao et al., 2021a; Cao et al., 2021b) (Figure 4).

### 2.3 Protective effect on liver injury induced by physical and chemical factors

#### 2.3.1 Radiation-induced liver injury

Ionizing radiation has been proven to lead to the imbalance of pro-oxidative and antioxidant activities through the production of ROS(Srinivasan et al., 2006). In one study, primary hepatocytes were isolated *in vitro* and treated with different doses of γ-irradiation (1, 2, 4 Gy) (Srinivasan et al., 2006). The results showed that with increasing γ-radiation dose, the level of TBARS and the severity of DNA damage increased markedly, and the activities of CAT, SOD and GPx decreased significantly. Compared with the model group, the expression of antioxidant enzymes in the FA treatment group was significantly increased, while the levels of DNA damage and TBARS were significantly decreased. These results suggest that FA pretreatment can protect hepatocytes from radiation damage(Srinivasan et al., 2006).

#### 2.3.2 Fluoride-induced liver injury

Fluoride is a vital trace element that spreads through the cell membrane and enters the soft tissue, causing soft tissue damage(Panneerselvam et al., 2013). Chronic fluoride intake will directly affect the biochemical function of the liver, resulting in hepatocyte necrosis, degenerative changes, liver hyperplasia, central lobular necrosis, inflammation and hepatocyte infiltration. It has been reported that FA protects male Wistar rats against fluoride-induced hepatotoxicity(Panneerselvam et al., 2013). In this study, hepatotoxicity was induced by oral administration of 25 mg/L fluoride for 12 weeks. Liver injury was evaluated by detecting pathophysiological indices, total protein content and histopathological changes. The results indicated that fluoride markedly reduced the body weight of rats, and the changes caused by fluorosis returned to the normal level after oral administration of FA. The histopathological results also showed that fluoride treatment could cause obvious abnormalities in liver structure compared with normal liver, including periportal vein, central lobule, focal parenchyma inflammation and necrotic area with portal vein hyperaemia. FA treatment markedly decreased the levels of lipid hydrogen peroxide, restored the levels of antioxidants, and alleviated liver injury(Panneerselvam et al., 2013).

#### 2.3.3 Cadmium-induced liver injury

Cadmium (Cd) is a highly toxic pollutant. Acute or chronic exposure to Cd causes serious damage to multiple organs(Sanjeev et al., 2019). Among them, the liver is one of the most vulnerable organs. Sanasam Sanjeev et al. found that subcutaneous administration of Cd (10 mg/kg) for 15 and 30 days could induce obvious hepatotoxicity, leading to liver parenchyma disorder, focal necrosis and hepatocyte swelling, while TNF-α, COX-2 and HSP70 proteins were markedly increased(Sanjeev et al., 2019). At the same time, the ratio of AST, ALT, ALP, LDH and the level of oxidative stress markers in the liver increased. Supplementation with FA (50 mg/kg) significantly reduced the concentration of Cd in liver tissue, protected the normal tissue structure of liver tissue, and restored serum total protein levels, serum NO levels and liver marker enzyme activity to normal levels(Sanjeev et al., 2019).

#### 2.3.4 Liver injury induced by aflatoxin b1

Aflatoxin b1, the most toxic mycotoxin in nature, has strong carcinogenicity to the liver (Rushing and Selim, 2019). Hepatotoxicity and carcinogenicity can only be produced after catalytic decomposition by a key enzyme (CYP450) *in vivo*(Rushing and Selim, 2019). One of the products of AFB1 catalysed by a specific CYP450 enzyme is aflatoxin 8d9-epoxide (AFBO) (Deng et al., 2018; Wang Z et al., 2021). The epoxides in the AFBO structure are highly reactive and can destroy the oxidation/antioxidation balance of cells. They covalently bind DNA and proteins in hepatocytes to form highly toxic metabolites, resulting in oxidative stress(Deng et al., 2018). Many studies have shown that AFBO inhibits the expression of antioxidant-related enzymes (SOD, GST, CAT, *etc.*), increases the level of oxidative stress markers (ROS, MDA, *etc.*, and causes oxidative damage, such as DNA fragmentation, apoptosis, and necrosis. AFBO combines with DNA to form an adduct AFB-N7-guanine (AFB1-DNA), which can form a stable structure by ring-opening, which can cause hepatocyte carcinogenesis when located in the transcriptionally active region of DNA. AFBO binds with serum albumin and hepatocyte proteins to form AFB1-lysine adducts, which can exist for a long time. The binding of AFBO to intracellular proteins can cause structural and functional disorders of proteins, leading to metabolic disorders, apoptosis, necrosis, and liver dysfunction(Deng et al., 2018). Glutathione S-transferase (GST) is a rate-limiting enzyme that catalyses the binding of AFBO with reduced glutathione (GSH) to form nontoxic metabolites. Therefore, inhibiting the key enzymes of CYP450 and activating GST, that is, hindering the metabolic activation of AFB1 or promoting the detoxification of AFBO, are the key targets to interfere with AFB1-induced oxidative stress in hepatocytes.

FA can inhibit AFB1-induced hepatocyte apoptosis and activate Nrf2/GST pathway. In one study, a liver injury model was established by feeding rats an AFB1 (300 μg/kg) diet, and an FA intervention group was established(Wang X et al., 2021). ALT, AST, AKP, GG and TBA levels in serum, ROS and MDA levels in the liver, pathological changes and apoptosis in liver tissues were further detected. The results showed that compared with the model group, hepatocyte necrosis was significantly reduced in the FA intervention group, and liver biochemical indices (GGT, TBA, ALT, AST and AKP) were also significantly decreased. Further investigation found that the expression levels of CYP450 protein in the FA intervention group were significantly reduced, and the Nrf2/GST pathway was activated. In addition, FA has been proven to inhibit hepatocyte apoptosis by regulating the expression of apoptosis-related proteins (Bax, Bcl-2, caspase-3) (Wang Z et al., 2021). FA can inhibit the production of AFBO, a highly toxic metabolite of AFB1, and reduce the oxidative damage of hepatocytes induced by AFB1 by inhibiting the expression of CYP1A2, CYP2A6, CYP2E1 and CYP3A4. On the other hand, it can interfere with AFB1-induced hepatocyte apoptosis and interfere with AFB1-induced liver injury by inhibiting the mitochondrial apoptosis signalling pathway(Wang Z et al., 2021).

## 3 Inhibition of liver fibrosis

Hepatic fibrosis (LF) is a traumatic repair response induced by various external stimuli that can cause a large amount of deposition of extracellular matrix (ECM), disorder of liver tissue structure and dysfunction of hepatocytes(Tsuchida and Friedman, 2017; Parola and Pinzani, 2019). LF is common in chronic viral hepatitis, nonalcoholic fatty liver and chronic alcoholism. As a necessary process for the development of liver cirrhosis, liver fibrosis can further develop into liver cirrhosis, liver cancer and liver failure, which is seriously harmful to human health. As early as 1979, Pérez-Tamayo R proposed in his literature that hepatic fibrosis is reversible(Perez-Tamayo, 1979).

The pathogenesis of hepatic fibrosis is very complex(Tsuchida and Friedman, 2017; Parola and Pinzani, 2019). An increasing number of studies have shown that the activation of HSCs is the most critical factor in the formation of hepatic fibrosis(Tsuchida and Friedman, 2017). Many inflammatory cells, cytokines and various cellular signal transduction pathways are involved in the progression of hepatic fibrosis. HSCs are distributed in the peri-sinusoidal space (Disse’s pace) of the liver next to liver sinusoidal endothelial cells (LSEC) and hepatocytes, accounting for 5%–8% of the total liver cells(Luo et al., 2021). HSCs are involved not only in the synthesis and degradation of the extracellular matrix but also in the regulation of hepatic sinusoid blood flow. Under normal physiological conditions, HSCs are in a resting state and hardly produce extracellular matrix. When the liver is subjected to various external stimuli and injuries, Kupffer cells (macrophages) in the liver can secrete and release a large number of cytokines(Yang et al., 2021). These cytokines stimulate and lead to the activation of HSCs. When HSCs are converted into myofibroblasts, they can synthesize a large amount of extracellular matrix, which is deposited in liver tissue and eventually leads to liver fibrosis. In the process of activation, HSCs can also release cytokines and further promote their own activation. These cytokines can also participate in the occurrence of liver fibrosis by binding to the corresponding receptors and activating the corresponding cellular signal transduction pathways. In addition, activated myofibroblasts can release matrix metalloproteinases (MMPs) and tissue inhibitors of metalloproteinase (TIMPs) (Tan et al., 2021). As the dynamic balance between MMPs and TIMPs is broken, the degradation of the extracellular matrix is inhibited. The continuous accumulation of collagen destroys the normal structure and liver function of the hepatic lobule. Portal pressure increases reduce the liver blood supply, leading to irreversible liver cirrhosis. At present, the main mechanisms of anti-fibrosis drugs are inhibition of liver inflammation and immune response, antioxidant injury, inhibition of HSC activation, inhibition of extracellular matrix (ECM) synthesis and acceleration of ECM degradation(Tan et al., 2021).

**FIGURE 5:**
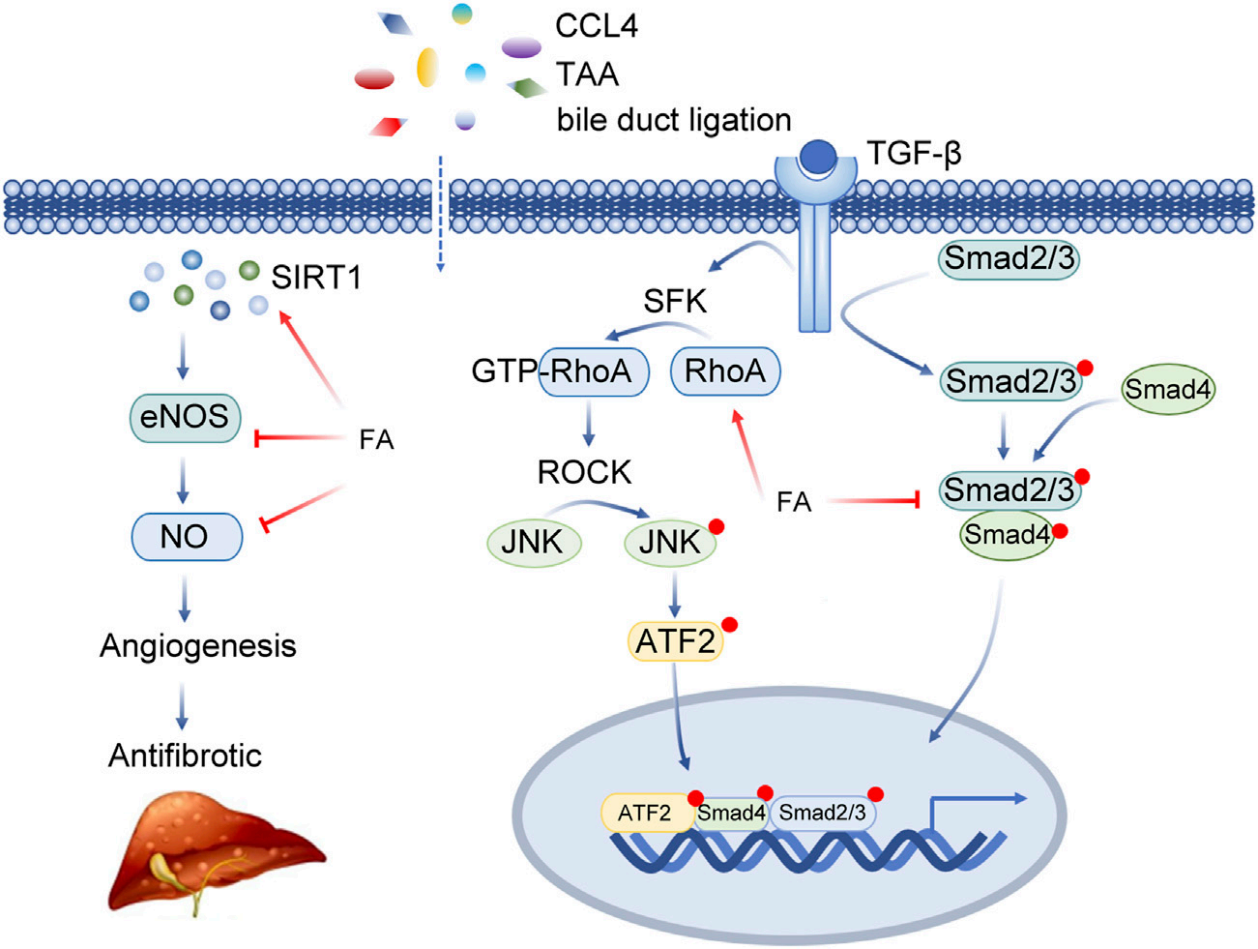
Insights into the multi-target action of ferulic acid in Liver fibrosis. Abbreviations: CCL4, carbon tetrachloride; TAA, thioacetamide; SIRT1, Sirtuin 1; eNOS, Nitric Oxide Synthase 3; NO, Nitric Oxide; TGF-β, Transforming Growth Factor Beta; SFK, SRC-Family protein tyrosine Kinase; RhoA, Ras Homolog Family Member A; ROCK, Rho Associated Coiled-Coil Containing Protein Kinase; JNK, Mitogen-Activated Protein Kinase 8; ATF2, Activating Transcription Factor 2; Smad2/3, SMAD Family Member 2/3; Smad4, SMAD Family Member 4.

FA inhibited ERK1/2 phosphorylation and reduced the expression of TGF-β1/Smad. Tianjiao Xu et al. systematically studied the effect of FA on HSCs through *in vitro* experiments (Table 2; Figure 5) (Xu et al., 2015). *In vitro*, FA dose-dependently inhibited the activity of HSC-T6 cells and decreased the expression of fibronectin and type I collagen. Further exploration of related signalling pathways revealed that FA inhibited ERK1/2 phosphorylation in a FAK-dependent manner. Meanwhile, FA treatment significantly reduced the expression of TGF-β1 and TGF-β receptors and inhibited the transcriptional activity of Smad(Xu et al., 2015).

### 3.1 Hepatic fibrosis induced by CCL4

The CC14-induced liver injury model is a classical model of liver injury(Dong et al., 2016). FA has been reported to reduce CCl4-induced liver fibrosis in rats, restore liver function, reduce liver tissue injury and suppress the degree of liver fibrosis(Wang Yuan-Yuan, 2021). Further studies confirmed that FA can exert antioxidant stress and antifibrotic inflammatory responses by inhibiting the activation of the MAPK and NF-κB pathways(Wang Yuan-Yuan, 2021).

Adenosine monophosphate activated protein kinase (AMPK), as a vital energy sensor, is regulated by phosphorylation of liver kinases (such as liver kinase B1) or dephosphorylation of protein tyrosine phosphatase (PTPs). Jianzhi Wu et al. reported that FA ameliorates CCl4-induced inflammatory and fibrotic liver injury in mice by reducing the activity of serum liver functional enzymes and inhibiting the expression of genes and proteins related to fibrogenesis(Wu et al., 2021). FA inhibited macrophage and hepatic stellate cell activation through the AMPK signalling pathway. In the absence of liver kinase B1(LKB1), the anti-inflammatory and antifibrotic effects induced by FA were counteracted by a specific AMPK inhibitor (Compound C). Combined with the results of molecular docking, surface plasmon resonance and coimmunoprecipitation experiments, it was further proven that FA directly binds and inhibits dephosphorylase protein tyrosine phosphatase 1 B (PTP1B), resulting in the phosphorylation of AMPK. Jianzhi Wu et al. confirmed that FA attenuated liver oxidative stress, liver inflammation and fibrosis through the PTP1B-AMPK signalling pathway(Wu et al., 2021).

Methyl ferulic acid (MFA) can effectively suppress the oxidative stress injury of the liver in rats. In another study, a hepatic fibrosis model was established by intragastric administration of 50% CCl4 olive oil solution(Li Chen, 2017). In the model group, the liver and spleen index increased. Meanwhile, the expression of procollagen type Ⅰ (PCⅠ) and α-SMA increased, which showed obvious characteristics of hepatic fibrosis. In the MFA groups, ALT, AST, procollagen type III peptide (PCIII), collagen type IV (IV-C), hyaluronic acid (HA), laminin (LN) and liver and spleen index decreased, and the expression of CAT, GSH-Px, α-SMA, PCI and SOD increased (Li Chen, 2017).

### 3.2 Hepatic fibrosis induced by TGF-β1

Transforming growth factor-β (TGF-β) is a cytokine with complex functions(Hu et al., 2018). The functions of TGF-β involve cell growth, extracellular matrix accumulation and immune response. In the TGF-β family, the TGF-β1-mediated signalling pathway inhibits apoptosis of HSCs and induces HSCs to synthesize excessive matrix proteins. Guo Ling et al. clarified the specific mechanism of FA in hepatic fibrosis by using primary rat hepatocytes(Guo Ling, 2019). The results indicated that FA suppressed the expression of Smad3 induced by TGF-β1, promoted the expression of MMP-2 and MMP-9, reduced hepatocyte apoptosis, promote hepatocyte proliferation and alleviate hepatic fibrosis(Guo Ling, 2019). Another study showed that the levels of type I collagen, α-SMA, fibronectin, and p-Smad in human HSCs (LX-2) treated with TGF-β1 were significantly increased, while FA treatment inhibited the abnormal increase in these proteins(Mu et al., 2018). Qi Cheng et al. found that 5 μg/L TGF-β1 induced fibrosis in HSC LX-2 cells, while MFA reduced the expression of α-SMA and type I collagen(Cheng et al., 2019a; Cheng et al., 2019b). Other studies also confirmed that FA restrains the activation of HSCs by inhibiting TGF-β1/Smad signal transduction(Xiong Meili, 2016). MFA (MFA) inhibits the proliferation of LX2 stimulated by TGF-β1, decreases the levels of α-SMA and PCI, and inhibits the synthesis of extracellular matrix and the transformation of the HSC-LX-2 phenotype(Xiong Meili, 2016).

### 3.3 Hepatic fibrosis in rats with bile duct ligation cirrhosis

Cholestatic liver disease is one of the major causes of human death(Jansen et al., 2017). Cholestatic cirrhosis is a pathological process characterized by bile duct hyperplasia, hepatocyte necrosis and liver fibrosis. At present, the mechanism of cholestatic cirrhosis is not fully clarified, and the treatment is limited. An animal model of common bile duct ligation is considered to be a relatively stable and effective method because of its similarity with the mechanism of cholestatic cirrhosis in humans. It has been confirmed that FA combined with astragaloside has a synergistic effect on hepatic fibrosis induced by bile duct ligation in rats(Zhao H et al., 2020). Juan Yang et al. also found that sodium ferulate has an antifibrotic effect on experimental hepatic fibrosis induced by bile duct ligation in rats(Juan Yang, 2014). The results of animal experiments showed that liver function was not significantly improved after treatment with sodium ferulate. However, pathological observation showed that the degree of hepatic fibrosis and hepatocyte destruction in the treatment group were lower than those in the model group. The results of immunohistochemistry showed that the expression of α-SMA in the liver of rats treated with sodium ferulate was lower than that of the model group(Juan Yang, 2014). In another study, a rat model of cholestatic hepatic fibrosis was established by bile duct ligation(Wei et al., 2014; Liu et al., 2015). It was proven that FA reduces the portal vein pressure of rats with cholestatic liver fibrosis after bile duct ligation (BDL) by interfering with the RhoA signalling pathway. In this study, compared with the sham operation group, the level of RhoA increased, while the expression of phosphorylated vasodilating-stimulating phosphoprotein (p-VASP), endothelial nitric oxide synthase (eNOS) and phosphorylated endothelial nitric oxide synthase (p-eNOS) decreased. After FA treatment, the expression of RhoA decreased, while the expression of p-VASP, eNOs, and p-eNOs increased(Wei et al., 2014; Zhao X M et al., 2020).

### 3.4 Hepatic maintenance induced by thioacetamide (TAA)

Thioacetamide (TAA) is a toxic substance(Kim et al., 2017). After entering the liver, it affects the metabolic process of related enzymes by prolonging hepatocyte mitosis and hindering RNA transfer, resulting in hepatocyte necrosis. Based on the above characteristics, TAA is often used to induce fibrosis in rodents(Kim et al., 2017). The model is very similar to the biochemical and morphological characteristics of human liver disease and is usually used as a suitable animal model for basic research of liver fibrosis. It has been reported that FA can significantly reduce the increase in serum ALT, AST and ALP activities induced by thioacetamide (TAA) and protect the integrity of liver tissue(Hussein et al., 2020). FA increased the activity of antioxidant enzymes in the liver and decreased the MDA content to the normal level. The total protein expression of TGF-β1, p-Smad3 and Smad3 in liver tissue decreased significantly. In addition, the expression of miR-21 was downregulated, and the expression of miR-30 and miR-200 was upregulated in the FA treatment group. In summary, FA has protective and antioxidant effects on TAA-induced hepatic fibrosis in rats by inhibiting the TGF-β1/Smad3 signalling pathway and differentially regulating the expression of microRNAs (miR-200, miR-30 and miR-21) in the liver(Hussein et al., 2020).

## 4 Inhibiting liver steatosis and reducing lipid toxicity

Adipose tissue has the physiological function of storing fat and is crucial for maintaining lipid metabolism in the body. However, in insulin-resistant patients, abnormal accumulation of free fatty acids in other organs, such as the liver, may cause lipotoxicity to the liver. Sirtuin1 (SIRT1) is a nicotinamide adenosine dinucleotide (NAD)-dependent deacetylase that mediates the removal of acetyl groups by a variety of proteins and plays an important role in maintaining liver lipid homeostasis.

FA inhibits the expression of inflammatory factors (IL-1β and IL-6) in a SIRT1/autophagy-independent pathway. An *in vitro* lipotoxicity model of mouse hepatocytes (AML-12) treated with palmitate was established(Xu et al., 2021). The levels of cell activity, apoptosis, mitochondrial function, autophagy and proinflammatory factors in each group were further detected, and the possible signalling pathways involved were further identified. The experimental results showed that FA reversed the hepatocyte apoptosis induced by palmitate, suppressed the excessive ROS generation induced by palmitate, and restored the mitochondrial membrane potential (MMP). FA increased the expression of the autophagy signature molecules LC3II and Beclin1 and upregulated the expression of Autophagy Related 5 (ATG 5) and ATG 7. FA-induced autophagy depends on SIRT1 activity(Xu et al., 2021).

Methyl ferulic acid (MFA) could downregulate JNK/MAPK/ERK signal pathway and relieve hepatic steatosis. Human hepatocyte-02 cells were induced by ethanol to establish an *in vitro* hepatic steatosis model(Cheng et al., 2018). By measuring the cell activity and lipid accumulation in each group, it was found that ethanol induced lipid droplets to accumulate in cells, while the total triglyceride (TG) and total cholesterol (TC) contents were significantly reduced after MFA treatment. MFA alleviated lipid deposition in cells. Further investigation showed that the protein levels of p-JNK, p-P38 MAPK and p-ERK were significantly decreased in the MFA treatment group. The protein levels of p-ACC and p-AMPK were significantly increased, and the mRNA levels of lipid oxidation-related genes (PPAR-α, SIRT1 and CPT-1α) were significantly increased in the MFA treatment group. Meanwhile, the animal models were established by feeding rats an ethanol-containing diet, and MFA (20/10/5 mg/(KGD)) intervention groups were set. Histopathological examination revealed that ethanol treatment resulted in an irregular arrangement of liver cells, partial hepatic steatosis, inflammatory cell infiltration and lipid droplet aggregation. The levels of high-density lipoprotein cholesterol (HDL-C) were significantly increased in the MFA treatment group, while TG and TC levels were significantly decreased. Meanwhile, the levels of p-FoxO1, p-AMPK and p-ACC were significantly upregulated in the MFA intervention group, and the levels of p-JNK, p-p38MAPK and p-ERK were significantly downregulated. Liver lipid deposition and hepatic steatosis were improved(Cheng et al., 2018). In another study, HepG2 cells were treated with 0.5 mM oleic acid (OA), and C57BL/6 mice were fed a high-fat diet (HFD, 5.28 kcal/g) to construct *in vitro* and *in vivo* models of steatosis(Wang Z et al., 2021). The results showed that liver degeneration, inflammation and swelling were improved in the FA intervention group. Further research showed that FA downregulated the expression of HMGB1 and reversed the intracellular lipid deposition induced by OA by inhibiting the JNK and ERK signalling pathways(Wang X et al., 2021).

## 5 Improving insulin resistance in the liver

Non-alcoholic fatty liver disease (NAFLD) is a serious threat to human health and is closely related to insulin resistance. In one study, the rat model of liver insulin resistance was established by feeding a liquid diet containing ethanol(Cheng et al., 2019a; Cheng et al., 2019b). The results showed that the indices of the liver and spleen of rats in the model group were increased, and a large amount of lipid droplet deposition and extensive necrosis occurred in liver cells, while MFA significantly suppressed liver injury. Further studies conclusively showed that MFA promoted the binding of PI3K-P110 and PI3Kp85, increased liver glycogen synthesis and played a protective role in alcohol-induced liver injury. At the cellular level, MFA promotes the expression of SREBP1 and p-FoxO1 and reduces the levels of PTEN, FBPase, PEPCK, G-6 Pase and TG. MFA increases glucose consumption and glycogen synthesis in liver cells and improve hepatic insulin resistance(Cheng et al., 2019a; Cheng et al., 2019b).

MFA was involved in the regulation of IR and P110α expression mainly through the mir-378b and PI3K-Akt pathways, thus improving the insulin sensitivity of the mouse liver. Previous studies have reported that mir-378b promotes insulin resistance by interfering with the expression of IR and P110 α(Zhang et al., 2022). Mir-378b expression was increased and IR/p-IR, p-IRS/IRS and p-Akt/Akt expression were significantly decreased in ethanol-treated L-02 cells. At the same time, the livers of ethanol-fed mice showed obvious vacuoles and steatosis, and the glucose tolerance of mice decreased. MiR-378b expression was significantly inhibited in the MFA group. Meanwhile, liver steatosis was significantly improved, and glucose tolerance was increased in the MFA group (Zhang et al., 2022).

## 6 Anti-liver cancer

Hepatocellular carcinoma (HCC) is a common malignant tumour. At present, the treatment of liver cancer is very limited. Conventional treatment can improve the short-term survival rates of patients, but recurrence rates are high. In recent years, metal oxide nanoparticles have received great attention because of their multipurpose properties and the importance of treatment(Ezhuthupurakkal et al., 2018). Some research groups combined FA with nano-chemotherapeutic drug carrier ZnO nanoparticles (ZnONPs) to prepare ZnONPs-fac(Ezhuthupurakkal et al., 2018). At the cellular level, ZnONPs-fac can inhibit the activity of hepatocellular carcinoma cells (Huh-7 and HepG2) in a dose-dependent manner, promote intracellular ROS production and induce DNA damage. At the same time, ZnONPs-fac can upregulate the expression of apoptotic proteins (Bax, Bad, cleaved caspase-3 and cleaved-PARP), downregulate the expression of anti-apoptotic proteins (Bcl-2 and Bcl-xL) and induce apoptosis of hepatocellular carcinoma cells. The results of animal experiments showed that the number of liver nodules and the levels of liver signature enzymes decreased significantly in the FA treatment group. FA significantly reduced liver injury, protected its normal structure and had good anticancer effects.

The main dilemma of anticancer therapy is resistance to drugs and radiation at the later stages of treatment, which may lead to the recurrence of the disease. Another study found that FA (FA) enhances the radiosensitivity of lung cancer (A549) and liver cancer (HepG2) cells(Das et al., 2019). Through the dual regulation of the redox state, FA improves the radiosensitivity of cancer cells and improves the effectiveness of radiotherapy(Das et al., 2019).

## 7 Other mechanisms

Hepatic ischaemia/reperfusion (I/R) is the main cause of liver injury after liver transplantation and resection and uncontrollable clinical conditions such as shock. I/R can further lead to liver dysfunction and distal organ injury. It has been reported that FA can attenuate the increase in serum transaminase activity, liver lipid peroxidation and liver glutathione content induced by I/R(Kim and Lee, 2012). FA can attenuate the excessive release of caspase-3 and cytochrome c induced by I/R, reduce the level of apoptosis-related proteins (Bax and tBid), inhibit p-JNK1/JNK2, and alleviate hepatocyte apoptosis induced by I/R(Kim and Lee, 2012). MMPs are a family of zinc endopeptidases that can degrade various ECM protein components and participate in tissue remodelling during fibrosis. In general, the degradation potential of MMPs is controlled by the tissue inhibitors of metalloproteinases. FA has been proven to significantly reduce the levels of collagen and TIMPs and regulate the expression of MMPs(Rukkumani et al., 2012). Oxidative stress is closely related to the development of various diseases. FA has been proven to suppress oxidative damage in the liver of adolescent diabetic rats(Thyagaraju, 2008). FA has also been confirmed to prevent diosbulbin B-induced liver injury by increasing the activity of CuZn-SOD and CAT(Wang et al., 2014).

## 8 Discussion

FA was first found in plant seeds and leaves. FA is a phenolic acid and it combines with polysaccharides and proteins in the cell wall to form the cytoskeleton of the cell wall, rarely in free form(Li C. et al., 2021; Li D. et al., 2021). It has a high content in ferulae, cohosh, Angelica sinensis, wild jujube seed and other traditional Chinese medicine, and it is one of the effective components of these traditional Chinese medicine. FA exists in edible plants such as onion and yellow chrysanthemum, and FA content is also high in grain hull, coffee, wheat bran, rice bran, bagasse, beet meal and other food materials. At present, the source of ferulic acid is mainly obtained from plants, and the production method is mainly alkaline hydrolysis. As a natural antioxidant, ferulic acid has pharmacological effects such as antioxidation, prevention and treatment of coronary heart disease and anticancer(Babbar et al., 2021). Its sodium ferulate is widely used in clinic. It has attracted more and more attention in combination with the treatment of nephropathy, pulmonary hypertension, cerebral infarction, Alzheimer’s disease and other drugs. Because ferulic acid has the function of anti-oxidation and scavenging free radicals, ferulic acid also has certain value and market development prospect in food, cosmetics and other fields. Various molecular mechanisms have been shown to be strongly related to FA in preventing and ameliorating various liver diseases, including reduction of liver oxidative stress injury, anti-inflammatory, anti-lipid peroxidation, inhibition of hepatocyte apoptosis, and promotion of autophagy. These important findings advance our knowledge of FA in the treatment of various liver diseases, indicating that FA has broad application prospects in disease control(Babbar et al., 2021).

FA has been shown to be effective in the treatment of many liver diseases in recent years, but there are still insufficient clinical data. Researchers previously treated 28 patients with cirrhosis of the liver with sodium ferulic acid in combination with ligustrazine(Li Yunhua, 2006). The study found that endothelin is the strongest vasoconstricting peptide, and the contractile vein is significantly more effective than the artery, increasing portal blood flow resistance and portal pressure and worsening liver microcirculation disorders during cirrhosis. At the same time, endothelin directly damages hepatic vascular endothelial cells, activates various growth factors and promotes liver fibrosis. Sodium ferulic acid, an endothelin receptor antagonist, was used to treat patients with sodium ferulic acid, which resulted in lower endothelin levels and significantly improved liver fibrosis(Li Yunhua, 2006). This study confirmed that the addition of sodium ferulic acid and ligustrazine intravenous drips to routine liver preservation therapy protected liver cells, improved liver function, protected vascular endothelial cells, improved liver microcirculation, and slowed liver fibrosis(Li Yunhua, 2006). Although these clinical trials have shown that FA can improve cirrhosis of the liver, no studies have been reported on its use in the treatment of other clinical conditions. Therefore, more clinical trials are needed in the future to assess the hepatoprotective role of FA in different disease models.

FA was determined to have low toxicity, considering absorption, excretion and lethal doses(Yan et al., 2020). Due to its low stability, poor lipophilicity, polarity and nonpolarity imbalance, its application in food, cosmetics and other fields is still limited. Therefore, advanced drug delivery systems are being explored to deliver sufficient concentrations of therapeutic drugs to the circulatory system and target tissues to improve their bioavailability and practical applications(Grimaudo et al., 2020). Future studies should further elucidate the pharmacokinetics of FA *in vivo*, especially in the human liver, to enhance the efficacy of FA in the treatment of various diseases. At the same time, we also need to clarify the compatibility of drugs.

While a large number of studies have elucidated signalling pathways and molecules that FA may play a role in, in future studies, we can further improve the accuracy and effectiveness of disease treatment by screening better targeted molecules in combination with a variety of histochemical approaches, such as metabolomics, transcriptomics, and proteomics.
